# A complete chloroplast genome sequence of *Gastrodia elata* (Orchidaceae) represents high sequence variation in the species

**DOI:** 10.1080/23802359.2019.1710588

**Published:** 2020-01-14

**Authors:** Jongsun Park, Youngbae Suh, Sangtae Kim

**Affiliations:** a Infoboss Co., Ltd, Gangnam-gu, Seoul, Korea;; b InfoBoss Research Center, Gangnam-gu, Seoul, Korea;; c Natural Products Research Institute, Seoul National University, Seoul, Korea;; d Department of Biology, Sungshin University, Seoul, Korea

**Keywords:** *Gastrodia*, chloroplast genome, *Gastrodia elata*, intra-specific variation, Orchidaceae

## Abstract

*Gastrodia elata* is a non-photosynthetic saprophytic plant of medicinal use in the oriental countries. We report the second complete chloroplast (cp) genome of *G. elata* from a sample collected in Korea. The length of cp genome is only 35,180 bp: there is no inverted repeated region and many photosynthesis genes are missing compared to typical angiosperm cp genomes. It includes 20 protein-coding genes, 3 rRNAs, and 5 tRNAs. The overall GC content of the genome was 26.7%. Relatively, high intra-specific variation (457 SNPs and 670 indels) is detected in the species comparing it with other seed plants.

*Gastrodia elata* Blume is a perennial saprophytic species in Orchidaceae. It is distributed in South and East Asia including Nepal, Bhutan, India, China, Taiwan, Japan, and Korea. Stomach-shaped underground rhizome of this plant has been used in traditional oriental medicine, especially for treating headaches, dizziness, tetanus, and epilepsy (Tsaia et al. 2011).

Total genomic DNA was extracted from fresh leaves of *G. elata* (voucher: *S. Kim 2019-115* deposited in the Herbarium of the Sungshin University; N33°18′02.94″, E126°35′31.65″; Is. Jeju, Korea) by using a DNeasy Plant Mini kit (QIAGEN, Hilden, Germany). Genome sequencing was performed using HiSeq4000 at Macrogen Inc. and *de novo* assemblies have performed using both Velvet 1.2.10 (Zerbino and Birney 2008) and SOAPGapCloser (Zhao et al. 2011) under the in-house bioinformatics pipeline. Geneious R11 11.0.5 (Biomatters Ltd., Auckland, New Zealand) was used for genome annotation based on previously reported *G. elata* chloroplast (cp) genome (NC_037409; Yuan et al. 2018).

The length of the assembled cp genome of *G. elata* (Genbank accession number: MN296709) is 35,810 bp. In comparison to the previous report on cp genome from the Chinese plant of *G. elata* (NC_037409; Yuan et al. 2018), there are no major structural changes in this genome. As previously reported in *G. elata*, many genes related to photosynthesis are also missing and there are no inverted repeat regions. It contains 28 genes containing 20 protein-coding genes, 3 rRNAs, and five tRNAs. The overall GC content of *G. elata* was 26.7%.

Infra-specific cp genome variation in *G. elata* is considerably high compared to those in other Orchidaceae species. Four-hundred and fifty-seven single nucleotide polymorphisms (SNPs; 1.27%) and 670 insertions and deletions (INDELs; 1.87%) are identified. In another case of the Orchidaceae, *Goodyera schlechtendaliana* (LC085346 and NC_029364; Oh et al., 2019) showed 700 SNPs (0.45%) and 2133 INDELs (1.38%). Infra-specific cp genome variation in Orchidaceae is significantly higher than those in other seed plants: 121 SNPs (0.076%) and 781 INDELs (0.49%) in *Pyrus ussuriensis* (Rosaceae; Cho et al. 2019); 48 SNPs (0.031%) and 58 INDELs (0.037%) in *Duchesnea chrysantha* (Rosaceae; Park, Kim, Lee 2019); 93 SNPs (0.060%) and 56 INDELs 0.036%) in *Abeliophyllum distichum* (Oleaceae; Park, Kim, Xi, Jang, et al. 2019); 84 SNPs (0.056%) and 125 INDELs (0.084%) in *Pseudostellaria palibiniana* (Caryophyllaceae; Kim et al. 2019); 78 SNPs (0.050%), and 643 INDELs (0.41%) in *Camellia japonica* (Theaceae; Park, Kim, Xi, Oh, et al. 2019); 21 SNPs (0.015%) and 114 INDELs (0.080%) in *Illicium anisatum* (Illiciaceae; Park, Kim, Xi 2019); 84 INDELs (0.054%) in *Coffea Arabica* (Rubiaceae; Park, Kim, Xi, Heo 2019).

Phylogenetic analysis was performed for 41 previously reported cp genomes in Orchioieae and Epidendroideae including the cp genome of the current study and seven outgroup taxa in Cypripediodeae using nine conserved chloroplast genes (*rpl20*, *ycf2*, *rps18*, *rps8*, *rpl16*, *rps7*, *clpP*, *rps19*, and *rpl14*). The tree showed that that two *G. electa* cp genomes were placed together to form a clade, which is nested in the clade of Orchioieae and Epidendroideae with a long autapomorphic branch (Figure 1). *Calanthe triplicate* and *C. davidii* were sister groups to the clade of *G. eleata*.

**Figure 1.**
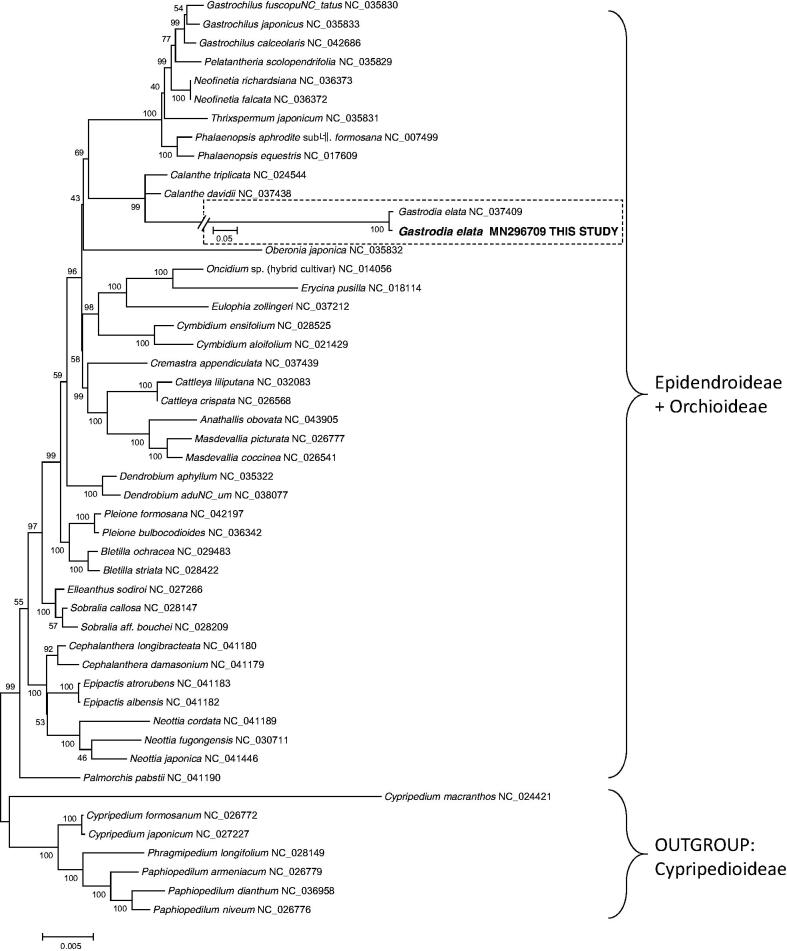
A maximum-likelihood tree based on “GTR + gamma + I” model using MEGA X (Kumar et al. 2018). Outgroup taxa were selected based on Górniak et al. (2010). The dashed box indicates a different-scaled region from the main tree. Numbers above the nodes indicate supporting values in 500 replicates of bootstrap analysis.
